# Anti-Tobacco

**Published:** 1892-10

**Authors:** 


### ANTI-TOBACCO.

Trask, the anti-tobacco apostle of the last generation, sowed a great deal of good seed in his pungent tracts, reaping much ridicule and little encouragement. But his party is growing. We are glad to learn that a movement has been inaugurated in France in opposition to the tobacco habit, which should be followed in this country, and, indeed, in all countries where the filthy and senseless habit prevails. In a recent number of the journal of the Society it is shown that a most vigorous and active work is being done by it.

